# A Role for Amyloid in Cell Aggregation and Biofilm Formation

**DOI:** 10.1371/journal.pone.0017632

**Published:** 2011-03-08

**Authors:** Melissa C. Garcia, Janis T. Lee, Caleen B. Ramsook, David Alsteens, Yves F. Dufrêne, Peter N. Lipke

**Affiliations:** 1 Biology Department, Brooklyn College of the City University of New York, Brooklyn, New York, United States of America; 2 Université catholique de Louvain, Institute of Condensed Matter and Nanosciences, Louvain-la-Neuve, Belgium; University of Minnesota, United States of America

## Abstract

Cell adhesion molecules in *Saccharomyces cerevisiae* and *Candida albicans* contain amyloid-forming sequences that are highly conserved. We have now used site-specific mutagenesis and specific peptide perturbants to explore amyloid-dependent activity in the *Candida albicans* adhesin Als5p. A V326N substitution in the amyloid-forming region conserved secondary structure and ligand binding, but abrogated formation of amyloid fibrils in soluble Als5p and reduced cell surface thioflavin T fluorescence. When displayed on the cell surface, Als5p with this substitution prevented formation of adhesion nanodomains and formation of large cellular aggregates and model biofilms. In addition, amyloid nanodomains were regulated by exogenous peptides. An amyloid-forming homologous peptide rescued aggregation and biofilm activity of Als5p^V326N^ cells, and V326N substitution peptide inhibited aggregation and biofilm activity in Als5p^WT^ cells. Therefore, specific site mutation, inhibition by anti-amyloid peturbants, and sequence-specificity of pro-amyloid and anti-amyloid peptides showed that amyloid formation is essential for nanodomain formation and activation.

## Introduction

*Candida albicans* is a human commensal fungus that is pathogenic when its growth becomes uncontrolled, especially in imunocompromised individuals. Under such conditions these eukaryotes can form biofilms that are resistant to a variety of environmental assaults, including antimicrobials [1], [2], [3]. The formation of biofilms is a developmental process with multiple steps including cell adhesion, extracellular matrix production and the formation of hyphae [4], [5].

**Figure 1:**
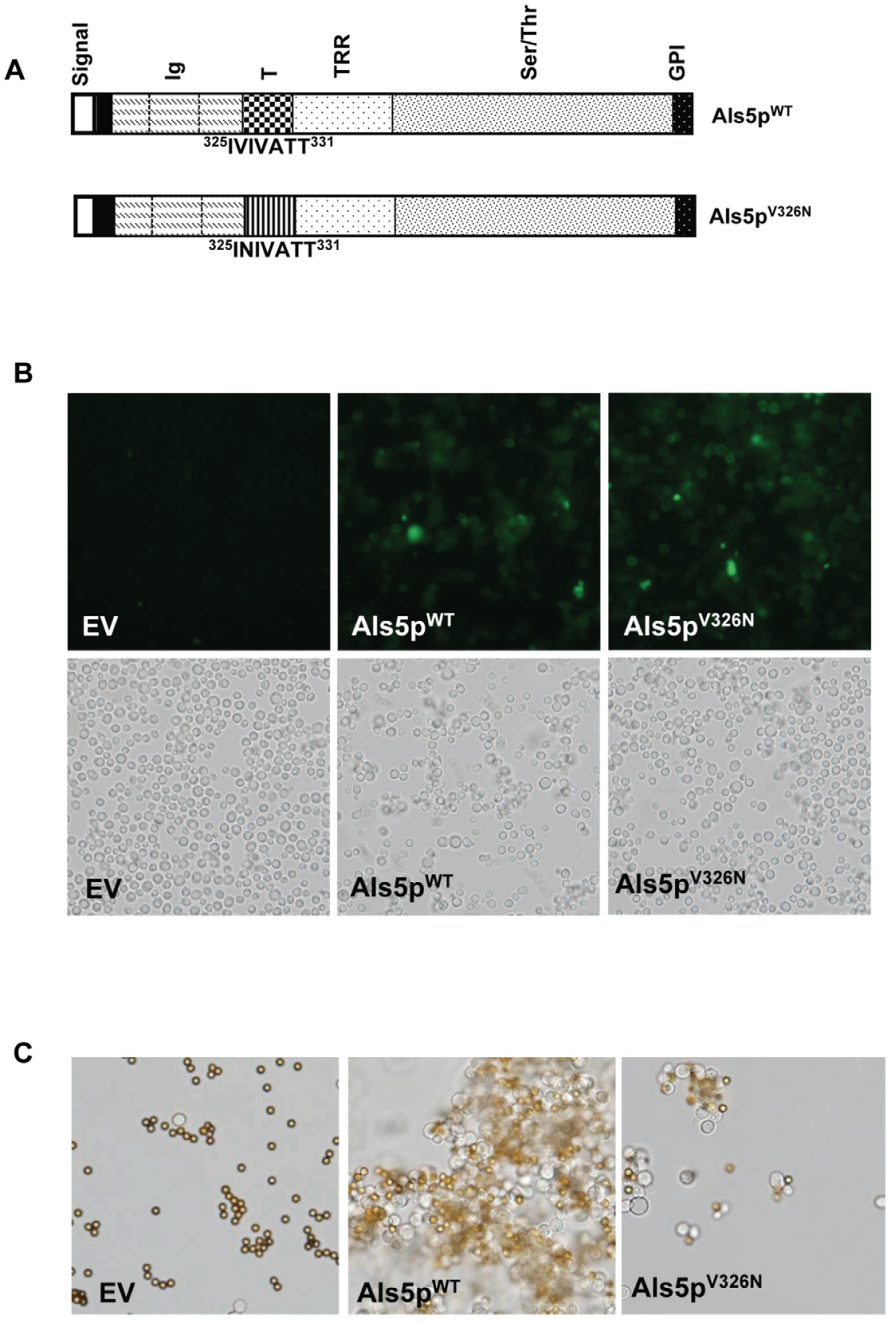
Effect of V326N mutation on Als5p expression and aggregation in *Saccharomyces cerevisiae.* (A) Maps of Als5p^WT^ and Als5p^V326N^. The open reading frame is 1419 amino acid residues long, and the mutation is near the N-terminus of the 103-residue T domain. (B) Immunofluorescence analysis shows expression of V5-tagged Als5p^WT^ and Als5p^V326N^ in *S. cerevisiae.* (C) Adherence and aggregation of yeast cells (gray) to heat denatured BSA-coated beads (brown-gold) for *S. cerevisiae* without Als5p (EV) or expressing Als5p^WT^ or Als5p^V326N^. Images shown were visualized using bright field microscopy. The diameter of the beads is 2.8 µm.

Cell wall proteins called adhesins are critical for biofilm formation, and mediate adhesion of *C. albicans* to various substrates and each other. Among many adhesins, members of the Als family of glycoproteins are particularly active in cell aggregation, adhesion to endothelia and epithelia, formation of biofilms and pathogenesis in mouse models. There are eight *ALS* gene loci, with high heterozygosity, so Als adhesin sequences and binding specificities are diverse. However, all Als proteins have similar modularity and domain structure (Figure 1A) [6], [7]. Three N-terminal Ig-like domains determine substrate specificity [7], [8], [9]. A 103-residue Thr-rich T domain is highly conserved among paralogs, and contains a 7-residue sequence that forms amyloids under native–like conditions [10], [11]. The central region of the protein contains a variable number of tandem repeats (TR domains) that are 36 amino acids in length, and these repeats bind to each other and to substrates through the hydrophobic effect [12], [13]. A highly glycosylated, C-terminal, serine- threonine-rich (Ser/Thr) stalk precedes a GPI anchor, which is processed to form a covalent linkage to cell wall polysaccharide [14]. The overlapping binding specificities and variable expression of Als proteins make them difficult to study in *C. albicans* [6], [7], [9], [15]. Nevertheless, expression in *Saccharomyces cerevisiae* has shown that Als1p, Als3p and Als5p bind similarly to a variety of substrates and are involved in endothelial cell adhesion, fungal aggregation, and tissue invasion [7], [16], [17], [18], [19].

Als-mediated adhesion to substrate is followed 5–20 min later by aggregation of Als-expressing yeast cells [20]. This aggregation is accompanied by a cell surface conformational change in the Als proteins to mediate stronger adhesive interactions [13], [21]. This activation is independent of cellular metabolism, because it occurs in heat-killed cells [13].

The T domains of Als proteins contain amyloid-forming sequences that are highly conserved [10]. Amyloids are insoluble fibrillar protein aggregates whose cores consist of crystalline arrays of identical sequences in many molecules of the amyloid protein [22], [23]. In the presence of amyloids, Congo red absorbance is red-shifted and increased, and the fluorescence emission of thioflavin T increases several fold [24], [25], [26]. At high concentrations, these dyes can perturb amyloid structure [27], [28], [29]. Some amyloid-forming bacterial adhesins can elicit cell-cell and cell-substrate adhesion leading to the formation of biofilms [30], [31], [32]. Other known roles for amyloid include amyloid-like stacking of residues in β-helices in viral spike proteins [33], curli in gram negative bacteria [30], [32], [34], sequestration of regulatory proteins in yeast [35], packing of pro-hormones in secretory vesicles [36], and as template activity for melanin assembly [37].

Previously, we showed that the amyloid sequence in the T region of Als proteins mediates amyloid formation, and that amyloid binding dyes can inhibit aggregation in the *S. cerevisiae* surface display model [8], [10], [11]. However, there is no direct data for the roles of Als amyloid sequences per se *in vivo*. We have therefore created a version of Als5p with its amyloid sequence disrupted by a single site substitution, and assayed its effects in the *S. cerevisiae* display model. We report the effects of this mutation on aggregation and biofilm formation in a model system. In addition, we have tested amyloid-forming and amyloid-inhibiting peptides for their effect *in vivo* in *C. albicans.* Finally, we provide direct evidence for the formation of amyloid adhesion nanodomains in *C. albicans,* using single-molecule atomic force microscopy (AFM) [38].

## Results

### Cells expressing a mutation in the amyloid sequence of Als5p exhibit less efficient aggregation

*S. cerevisiae* cells expressing Als5p form large aggregates, similar to those seen in *C. albicans* [8], [39]. This cell aggregation can be inhibited by amyloid binding dyes [8], [11]. A V326N mutation in the Als5p sequence reduces TANGO β-aggregation potential of the amyloid region from 93% to 4% [10]. To test the hypothesis that the amyloid forming sequence is critical for cell aggregation, we incorporated the V326N mutation into full length wild type Als5p, and expressed it in *S. cerevisiae* (Figure 1A). Immunofluorescence confirmed that that both wild type Als5p (Als5p^WT^) and the substitution sequence (Als5p^V326N^) were expressed on the yeast cell surface, with non-amyloid Als5p^V326N^ expression slightly greater than Als5p^WT^ (Figure 1B). In agreement with previous results, cells expressing Als5p^WT^ bound to heat denatured BSA-coated beads and formed large aggregates (Figure 1C) [8], [11], [39]. Cells expressing Als5p^V326N^ also bound to most of the beads, but formed much smaller aggregates. *S. cerevisiae* with vector alone did not bind to beads or form aggregates. Therefore, the V326N substitution had a small effect on binding BSA as a ligand, but a compromised the ability of Als5p to mediate formation of yeast aggregates.

**Figure 2:**
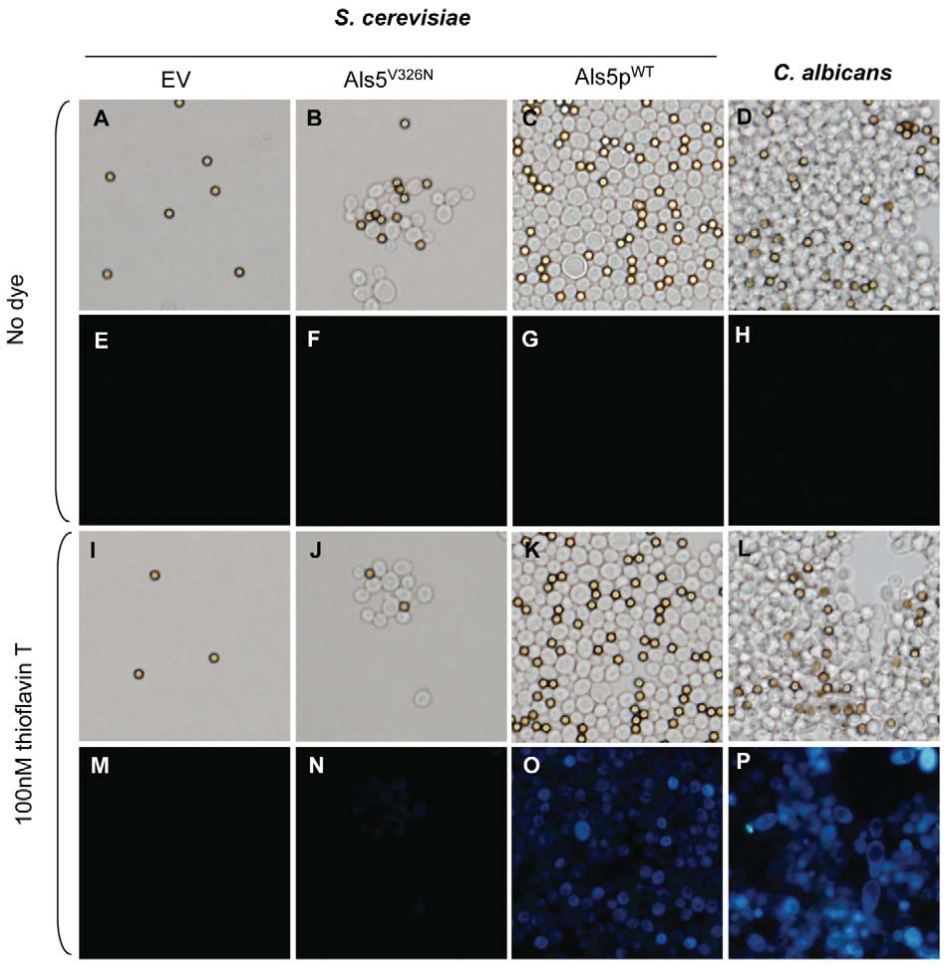
Thioflavin T fluorescence of *S. cerevisiae* and *C. albicans*. *S. cerevisiae* expressing no Als5p (EV), Als5p^V326N^, Als5p^WT^, or *C. albicans* were aggregated in the (A–H) absence or (I–P) presence of 100 nM thioflavin T. Aggregation state (upper panels) and thioflavin T fluorescence (lower panels) were monitored. The diameter of the beads is 2.8 µm, and all images are at the same magnification.

Increased thioflavin T fluorescence is a characteristic test for amyloid formation [40]. Aggregated *C. albicans* or Als5p^WT^ *S.cerevisae* cells stained brightly with 100 nM thioflavin T (Figure 2O and 2P), a concentration that had no effect on cell aggregation (Figure 2A–2D *vs.* 2I-2L). In contrast the small aggregates of cells expressing Als5p^V326N^ showed little fluorescence under these conditions (Figure 2N). Therefore, thioflavin T fluorescence was associated with robust aggregation of cells expressing amyloid-forming adhesins.

**Figure 3 pone-0017632-g003:**
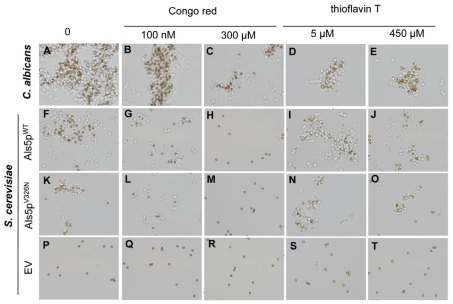
Effect of Congo red and thioflavin T on aggregation. (A–E) *C. albicans*, (F–J) Als5p^WT^ in *S. cerevisiae* or (K–O) Als5p^V326N^ in *S. cerevisiae*, and (P–T) empty vector (EV) cells were aggregated with beads coated with heat denatured BSA in the absence and in the presence of 100 nM or 300 µM Congo Red or 5 µM or 450 µM thioflavin T. Aggregates were observed by light microscopy. The diameter of the beads is 2.8 µm.

Greater concentrations of amyloid-binding dyes such as Congo red and thioflavin T inhibit aggregation of *S. cerevisiae* cells expressing Als proteins [11], [17]. If these dyes are acting through perturbation of the amyloid, they should not affect the adherence or residual aggregation of cells expressing Als5p^V326N^. We determined dye concentrations inhibiting cell aggregation and substrate binding for Congo red and thioflavin T (Figure 3). Both Als5p^WT^ and *C. albicans* showed inhibition of aggregation in the presence of 100nM Congo red (Figures 3A *vs.* 3B and 3F *vs.* 3G). This concentration had no visual effect on the ability of the Als5p^V326N^ to mediate formation of small aggregates (Figures 3K *vs.* 3L). Furthermore, this concentration is at least a thousand-fold lower than the concentrations that inhibit growth and fungal cell wall biogenesis [41]. A higher concentration of Congo red (300 µM) reduced the ability of both Als5p^WT^ and Als5p^V326N^ cells to bind to the beads and form aggregates (Figures 3H and 3M). Negative control cells did not aggregate with or without treatment (Figures 3P–3R).

Thioflavin T (5 µM and 450 µM) also decreased cell-cell adhesion in *C. albicans* (Figure 3A *vs.* 3D and 3E) and *S. cerevisiae* expressing Als5p^WT^ (Figure 3F *vs.* 3I and 3J). Thioflavin T had no effect on the smaller aggregates of *S. cerevisiae* Als5p^V326N^ cells (Figure 3N and 3O) or on empty vector cells (Figure 3S and 3T). These data support the idea that most Als-mediated aggregation is dependent on formation of amyloids.

### V326N soluble protein is deficient in amyloid formation and maintains native substrate binding activity

We expressed the V326N mutation in two soluble versions of the Als5p protein: Als5p^1-431^, including the Ig-like and Thr-rich amyloid region; and Als5p^1-664^, which also includes the tandem repeat region [8], [10], [12]. The proteins containing the V326N substitution were purified by published procedures and did not form amyloid fibers visible by electron microscopy (data not shown). When these soluble proteins were tested in modified ELISA assays for *in vitro* binding to fibronectin and polystyrene, the results were similar to those for the wild type Als5 protein within experimental error for both the 431-residue and 664-residue fragments. (Figure S1). Therefore, the *in vitro* binding properties of the proteins were not significantly altered by the substitution. Far UV Circular Dichroism spectra and secondary structure were also similar to those from the corresponding proteins with wild type sequences (Figure S2). These results demonstrated that the V326N substitution did not significantly affect ligand binding properties or secondary structure of Als5p.

### An amyloid-forming peptide restores aggregation of non-amyloid-forming cells

**Figure 4 pone-0017632-g004:**
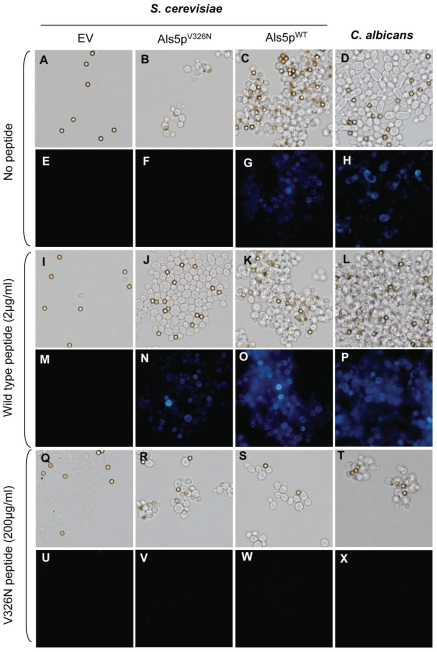
Effects of amyloid and non-amyloid peptides on cellular aggregation and thioflavin T fluorescence. *S. cerevisiae* expressing no Als5p (EV), Als5p^V326N^, Als5p^WT^, or *C. albicans* were aggregated in the (A–H) absence, or (I–P) presence of amyloid forming or (Q–X) amyloid-disrupting peptides. The amyloid-forming peptide was SNGIVIVATTRTV, and the amyloid-disrupting peptide was SNGINIVATTRTV. Each vertical pair shows brightfield and thioflavin T fluorescence images of the same field. The diameter of the beads is 2.8 µm, and all images are at the same magnification.

A tridecapeptide consisting of the sequence of Als5p^WT^ residues 322–334 (SNG**IVIVATT**RTV, amyloid sequence bolded) rapidly forms insoluble amyloids [10]. We reasoned that its strong amyloid-forming ability might increase amyloid formation in non-amyloid Als5p^V326N^ by providing a stable amyloid template. *S. cerevisiae* expressing either form of Als5p and *C. albicans* were incubated without (Figure 4A–4H) or with this wild-type sequence tridecapeptide (2 µg/ml) (Figure 4I–4P) during the bead assay. This peptide greatly increased aggregate formation in *S. cerevisiae* expressing Als5p^V326N^ (Figure 4B *vs.* 4J). The peptide had no detectable effect on the aggregation of empty vector, or Als5p^WT^-expressing *S. cerevisiae* or on *C. albicans* strains (Figure 4A *vs.* 4I, 4C *vs.* 4K and 4D *vs.* 4L). A scrambled-sequence peptide with the same amino acid composition as the Als5p amyloid-forming sequence (VITGVTNIRTSVA) did not induce aggregation, indicating that the observed effect was specific to the wild type sequence (Figure S3).

To determine if the peptide-induced adhesion was accompanied by formation of amyloid-like interactions, cells were stained with thioflavin T (Figures 4E–4H *vs.* 4M–4P). The small Als5p^V326N^ aggregates lacked intense thioflavin T fluorescence (Figure 4F). In contrast, aggregates formed from Als5p^V326N^-expressing cells in the presence of peptide exhibited more intense fluorescence (Figure 4N). In the presence of peptide, there was also increased fluorescence of the aggregates formed from *C. albicans* or Als5p^WT^-expressing *S. cerevisiae* (Figure 4G *vs.* 4O and 4H *vs.* 4P). Thus, exogenous amyloid-forming homologous sequence peptide induced increased aggregation in non-amyloid cells, and increased amyloid fluorescence in aggregating cells as well.

### Non-amyloid V326N peptide blocks aggregation

Since the amyloid-forming peptide potentiated aggregation of the Als5p^V326N^-expressing strain, we hypothesized that the mutant peptide would block cell aggregation. All strains were incubated with the V326N peptide (SNGI**N** ^326^IVATTRTV; 200 µg/ml) during the bead assay (Figure 4). This peptide strongly inhibited aggregation, including Als5p^WT^-expressing *S. cerevisiae* and *C. albicans* (Figure 4C *vs.* 4S and 4D *vs.* 4T). The few remaining aggregates looked similar to those observed with Als5p^V326N^ (Figure 4B). This peptide did not have an effect on either empty vector or Als5p^V326N^ strains (Figures 4A *vs.* 4Q and 4B *vs.* 4R). A scrambled V326N peptide (VITGNTNIRTSVA) did not block aggregation indicating that the inhibitory effect is specific to the V326N sequence (Figure S3).

Given that the V326N peptide blocked formation of aggregates in *C. albicans* and Als5p^WT^-expressing *S. cerevisiae*, we tested whether it prevented formation of amyloid-like interactions. In these strains, thioflavin T fluorescence was strongly reduced in the presence of V326N peptide (Figure 4G and 4H *vs.* 4W and 4X). The peptide had no effect on the fluorescence of the empty vector and Als5p^V326N^ aggregates (Figure 4U and 4V). These results show that the V326N peptide blocked amyloid formation and aggregation mediated by Als5p in *S. cerevisiae,* as well as aggregation in *C. albicans.*

### The amyloid sequence of Als5p is critical for cell-cell association and cell–substrate adhesion to polystyrene

**Figure 5 pone-0017632-g005:**
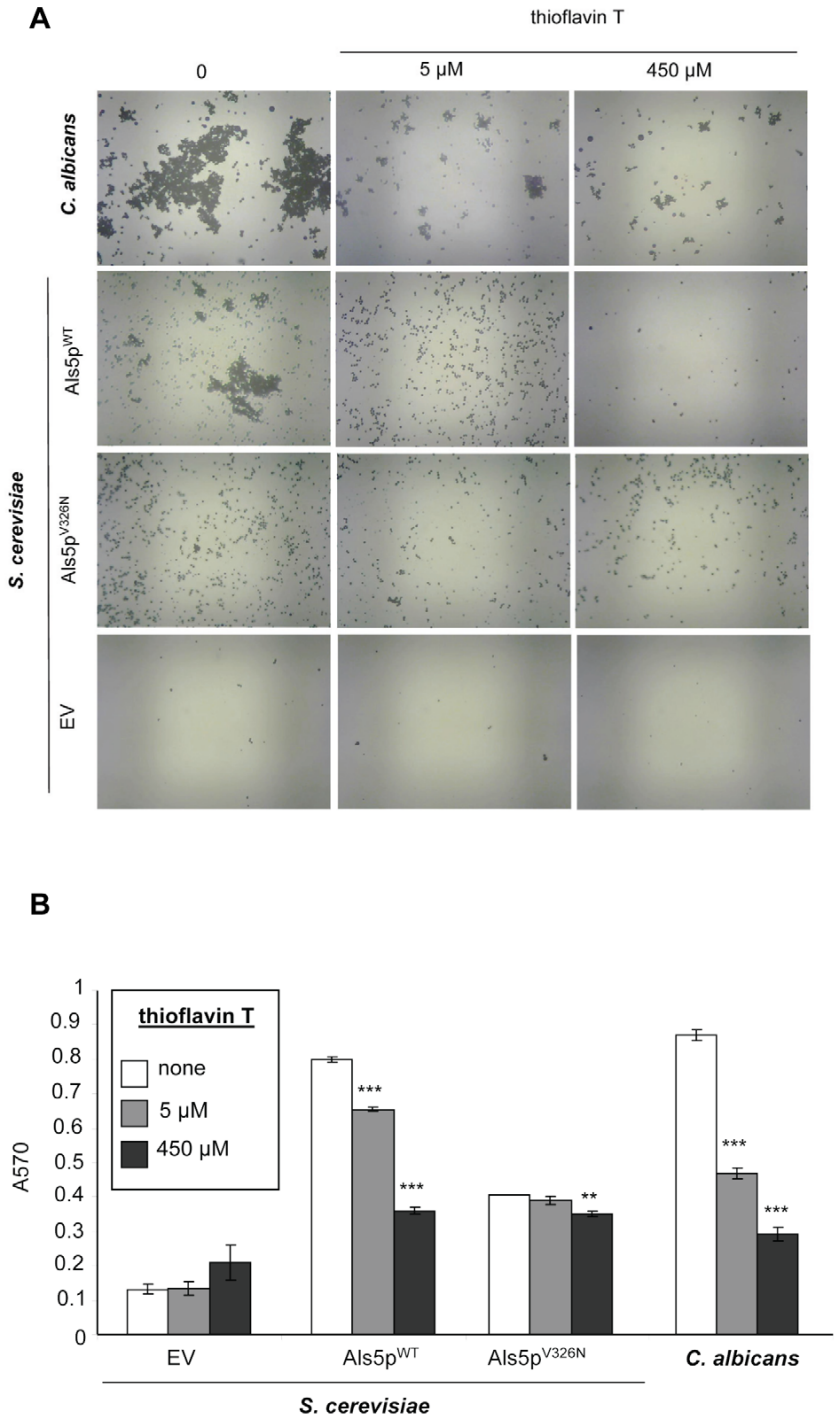
Effects of thioflavin T on binding and aggregation on a polystyrene surface. C. *albicans* or *S. cerevisiae* expressing Als5p^WT^, Als5p^V326N^, or empty vector (EV) strains were adhered to a polystyrene surface in the absence and presence of 5 µM or 450 µM thioflavin T for 1.5 h. Adherent cells were grown overnight, washed, stained with 1% crystal violet and imaged. (A) The bottoms of wells were imaged. (B) Triplicate biofilms were quantified with crystal violet. Mean and standard deviation are shown. Significance was determined by Student's T-test with p values relative to untreated strains (p≤0.05(*), p≤0.01(**), and p≤0.001(***)).

We also tested to determine whether amyloid formation was important for a model biofilm. *S. cerevisiae* expressing Als5p^WT^ or *C. albicans* adhered to the polystyrene surface of a 96-well plate in Tris-EDTA buffer. The non-adherent cells and buffer were removed, and the adhering cells incubated in medium overnight [42]. Microscopy and quantitative crystal violet staining of the wells revealed that Als5p^WT^-expressing *S. cerevisiae* and *C. albicans* strains bound to the surface in aggregates (Figure 5A). Thioflavin T (5 µM) partially dispersed the *C. albicans* aggregates and fully dispersed the Als5p^WT^-mediated aggregates of *S. cerevisiae.* This thioflavin T treatment reduced adherence of Als5p-expressing *S. cerevisiae* by about 16%, and *C. albicans* adherence by 45% (Figure 5B). A 90-fold higher concentration of dye reduced adherence to the plastic by 79% and 90% respectively. Congo red had similar effects: 100 nM disrupted aggregates and partially inhibited adherence; 300 µM blocked almost all adhesion (Figure S4).

In contrast to cells expressing Als5p^WT^, Als5p^V326N^ cells bound to the surface as dispersed cells, rather than as aggregates (Figure 5A). The lower concentrations of the dyes did not affect the residual adherence to the plastic surface, and the higher concentrations partially blocked adherence. Taken together, these results show that adherence to polystyrene is followed by aggregation to form a biofilm, and is mediated by the amyloid-forming region of Als5p in the *S. cerevisiae* model. The similar behavior in *C. albicans* further supports the hypothesis that amyloid sequences in Als or other adhesins are important in biofilm formation.

### Effects of an amyloid-forming peptide and an amyloid inhibitory peptide on model biofilms

**Figure 6:**
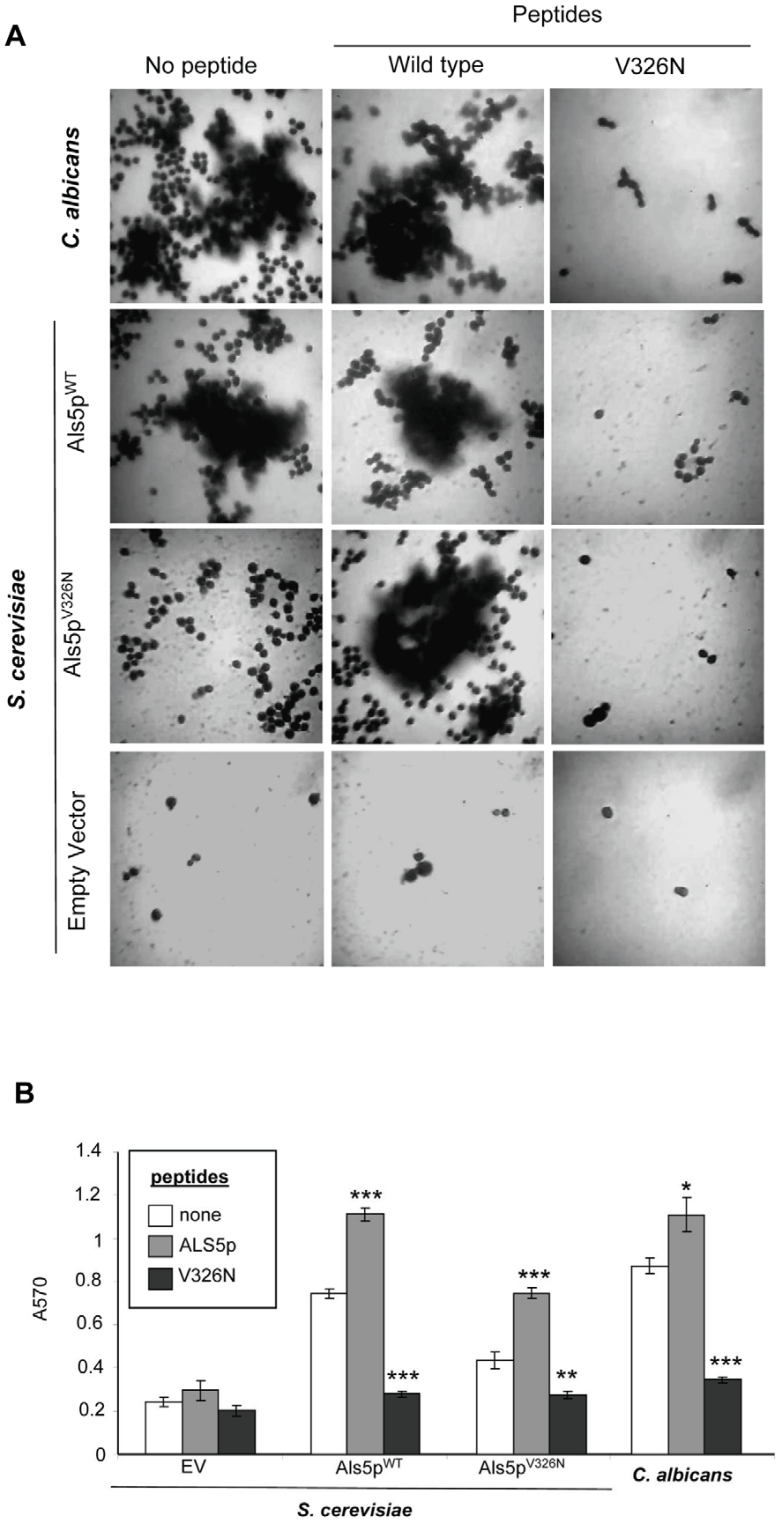
Effects of wild type and V326N peptides on polystyrene biofilms. (A) *C. albicans* or *S. cerevisiae expressing* Als5p^WT^, Als5p^V326N^, or no Als5p (EV) strains were adhered to a polystyrene surface in the absence and presence of the wild-type peptide (2 ug/ml) or V326N peptide (200 ug/ml) for 1.5 h. Adherent cells were grown overnight, stained with 1% crystal violet and imaged. (B) Crystal violet quantification of binding. Peptide-treated strains with p values relative to untreated strains were determined as significant by Student's T-test (p≤0.05(*), p≤0.01(**), and p≤0.001(***)).

Since a mutation in the Als5p amyloid-forming sequence disrupted adherence and aggregation on plastic, we hypothesized that the V326N and amyloid-forming Als5p peptides would also affect adhesion on polystyrene. The cells were incubated without or with amyloid forming peptide SNGIV^326^IVATTRTV or the V326N non-amyloid peptide. Microscopy and quantification revealed that the amyloid forming peptide rescued *S. cerevisiae* cells expressing Als5p^V326N^. In the presence of the amyloid-inducing peptide, these cells formed large aggregates like *C. albicans* and *S. cerevisiae* cells expressing wild type protein (Figure 6A). Conversely, the V326N peptide effectively blocked adherence and aggregation on plastic for each type of cell.

Quantification of adhesion to the polystyrene confirmed that Als5p^WT^-*S. cerevisiae* and *C. albicans* cells that were treated with amyloid-forming peptide bound better than untreated cells (Figure 6B). Additionally, Als5p^V326N^ cells that were treated with the wild type peptide exhibited a nearly two-fold increase in adherence relative to cells not incubated with peptide. These results show that a peptide inhibitor of amyloid formation can abrogate adherence to plastic and biofilm-like aggregation, and conversely that an activator of amyloid formation facilitates biofilm formation.

### AFM resolves Als adhesion nanodomains in *S. cerevisae* and *C. albicans* cells

**Figure 7:**
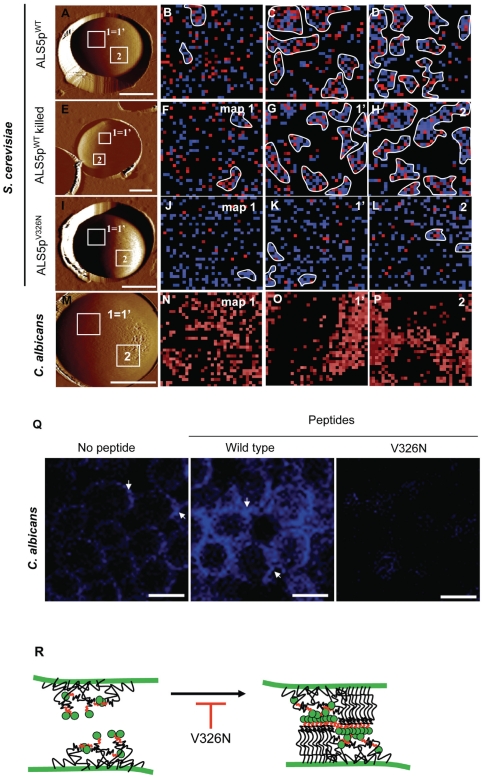
Force-induced adhesion nanodomains in single live cells. (A) AFM topographic image (scale bar: 2 µm), in buffer, showing wild-type *S. cerevisiae* cells expressing V5-tagged Als5p^WT^ proteins. (B) Adhesion force map (1 µm ×1 µm) recorded with an anti-V5 tip on a given target area of the native cell that was never subjected to force (maps #1, recorded on the square shown in (A). Blue and red pixels correspond to forces smaller and larger than 150 pN, respectively, thus to V5-tagged Als5p^WT^ recognition and unfolding. (C) Second adhesion force map (1 µm × 1 µm) recorded on the same target area (map #1′). The heterogeneous distribution of coloured pixels, which represents the detection of single Als5p^WT^ molecules documents the formation of nanoscale clusters (outlined in white). (D) Adhesion force map (1 µm × 1 µm) recorded on a remote area (map #2) localized several hundred nanometers away from the first map (see squares in A). (E–H) Same sequence of data as in A–D obtained on heat-killed *S. cerevisiae* cells expressing Als5p^WT^. (I–L) Same sequence of data as in A–D obtained on *S. cerevisiae* expressing ALS5p^V326N^. (M) AFM topographic image of a *C. albicans* cell (scale bar: 2 µm). (N) Adhesion force map (1 µm × 1 µm) recorded with an Als5p^1-431^-derivatized tip [44] on a given target area of the native cell (map #1, recorded on the square in M; red pixels correspond to unfolding forces that are in the 0-300 pN range). (O) Second adhesion force map (1 µm × 1 µm) recorded on the same target area (maps #1′). (P) Adhesion force map (1 µm × 1 µm) recorded on a remote area (map #2) localized several hundred nanometers away from the first map (see squares in M). (Q) Confocal imaging of punctate fluorescent nanodomains (white arrows) on aggregated *C. albicans* treated with Als5p or Als5p^V326N^ peptide and stained with 100 nM thioflavin-T. (R) Cartoon model of force-induced amyloid-dependent clustering of Als5p. Cell walls are shown as heavy greent lines. Als5p adhesion molecules have green Ig-like binding domains, a red amyloid sequence, and a black line representing the TR and stalk domains. Application of pulling force in the AFM or mixing in the aggregation assays causes formation of amyloid-like arrays on the cell surface. The formation of these arrays is blocked in the presence of Congo red, high concentrations of thioflavin T, or by V326N peptide or mutation.

Lastly, we used single-molecule AFM to probe the distribution of Als proteins in living *C. albicans* cells, with the aim to determine whether they are initially evenly distributed and clustered following application of force, as are Als proteins on *S. cerevisiae* [43](Figure 7). Yeast cells were trapped into porous polymer membranes, and analyzed using topographic imaging and spatially-resolved force spectroscopy. We first analyzed the *S. cerevisiae* surface display model expressing V5-tagged Als5p proteins using AFM tips bearing anti-V5 antibodies (Figure 7A–7L). Consistent with earlier work in *S. cerevisiae* [43] the initial distribution of Als5p^WT^ was random (Figure 7B). Pulling on single adhesins with the AFM tip induced the formation of adhesion domains of 100-500 nm size (Figure 7C). In addition, the force-induced nanodomains propagated over the entire cell surface, since remote areas showed similar nanodomains (Figure 7D). Als5p^WT^ remodelling was independent of cellular metabolic activity since heat-killed cells show the same behavior as live cells (Figure 7E–7H). Remarkably, Als5p clustering properties were almost completely abolished in the V326N mutant (Figure 7I–7L), indicating that amyloid interactions play a key role in clustering.

Because amyloid regions also appear in *C. albicans* (Figures 2–6) and are accompanied by development of strong adhesion and surface birefringence [8], [11], [20], we tested to see if there was similar force-induced formation of adhesin nanodomains in *C. albicans* (Figure 7M–7P). In this case, living *C. albicans* yeast cells were probed with AFM tips bearing Als5p^1-433^ fragments (Supplemental Figure S5A) since these bind to all Als adhesins [13], [16], [43], [44]. As expected, the initial force mapping showed a random pattern of Als proteins (colored pixels), with a surface density being greater than in the *S. cerevisiae* surface display model (Figure 7N vs. 7B). The adhesive forces were typical for Als-Als interactions (Figure S5B; [44]). Force-extension curves showed patterns similar to those reported earlier for Als5p-Als5p interactions [44], except that the number of unfolding Tandem Repeat domains varied from 3–33, the range known for Als alleles (Supplemental Figure S5C; [6]). Upon remapping of the same region (Figure 7, map1') or a remote region on the same cell (Figure 7, map 2), the adhesin molecules were clustered, as in the *S. cerevisiae* model. These observations lead us to conclude that nanodomains form and propagate in response to force in *C. albicans,* just as they do in Als5p^WT^-expressing *S. cerevisiae.*

Adhesion nanodomains are also visualized by confocal microscopy of thioflavin T-stained cells. Therefore we tested the effects of the peptides on nanodomain formation in *C. albicans.* As in the Als5p *S. cerevisiae* surface display cells, the native sequence peptide potentiated nanodomain formation and the V326N substitution peptide inhibited nanodomains. The sequence specificity of nanodomain potentiation and inhibition implies that Als proteins are major components of surface amyloids in *C. albicans.*

## Discussion

Amyloid-forming sequences are widespread in fungal adhesion proteins that form cellular aggregates [11]. That being said, there remains the question of whether amyloid formation *per se* is the function of these sequences *in situ* on the cell surface. We and others previously reported that anti-amyloid treatments disrupt Als protein-mediated aggregation [8], [10], [11], [17]. We have also demonstrated that a V326N mutation in Als5p prevents formation of surface nanodomains [43], but have not previously shown the functional consequences of this mutation. Therefore we set out to test directly the hypothesis that cell surface assembly of amyloids causes nanodomain formation and activation of adhesion. These new direct tests showed: first, that the V326N substitution in the amyloid core region of the intact cell surface-localized protein abrogated activation of adhesion; second, that the substitution did not affect secondary structure of the protein or its affinity for ligands; third, in agreement with the previous point, initial binding of cells to substrate was not affected by the substitution (Figures. 1, S1 and S2). We also took an independent and novel approach of monitoring effects of sequence-specific pro- and anti-amyloid peptides on nanodomain formation and adhesion activity. The effects were supportive of the hypothesis: the anti-amyloid peptide inhibited both nanodomain formation and adhesion activity, and the pro-amyloid peptide activated, even in cells expressing the V326N substituted protein. We also demonstrated for the first time that there are force-induced nanodomains on the surface of *C. albicans* and that the peptides perturb them. Finally, we showed that the peptides act similarly in a model biofilm, as do anti-amyloid dyes, a result also recently reported for Als3p by Nobbs et al. [17]. Together, these approaches confirm the hypothesis, showing that adhesion activity is increased by amyloid assembly and nanodomain formation. The widespread occurrence and the conservation of amyloid sequences in fungal adhesins are consistent with amyloid-mediated nanodomain formation being a general mechanism for activation of robust cell-cell aggregation.

We have tested this hypothesis by a detailed determination of phenotype for single site substitution V326N in the 1419 amino-acid Als5p sequence. This mutation is in the seven residue amyloid-forming sequence I^325^VIVATT^331^ in the highly conserved T domain of Als5p. The mutation reduces the TANGO β-aggregation potential, which is related to amyloid-forming ability, from >95% to 4% [45]. A peptide with this sequence change did not form amyloids *in vitro* [10]. Although 431-, and 664-residue fragments of Als5p^WT^ form amyloid fibers [10], [11], the corresponding soluble versions of Als5p^V326N^ did not (data not shown).

Therefore, the V326N mutation abrogated amyloid formation *in vitro* and also altered the activity of cell-bound Als5p. The mutation did not significantly affect cell wall localization of the protein (Figure 1), secondary structure (Figure S2), or binding of soluble Als5p to polystyrene or fibronectin (Figure S1). These results imply that the mutated protein folded to its native structure and retained its binding activity. Furthermore Als5p^V326N^ cell surface expression was consistent with proper folding and processing. Nevertheless, cells expressing Als5p^V326N^ bound to polystyrene or to heat denatured BSA-coated beads somewhat less efficiently than cells expressing the wild type protein (Figures 1, 5, and 6). However, there was a severe inhibition of cellular aggregation ability for cells expressing Als5p^V326N^. Thus the cellular consequences were great, despite the observation that the mutation did not affect *in vitro* activities of the soluble proteins. These consequences can be most easily explained as a failure of the mutated protein to form functional amyloid regions required for cell surface activation through adhesion nanodomain formation.

If the loss of amyloid formation is the key defect in Als5p^V326N^, then chemical inhibition of amyloid formation should show similar effects, and the residual activity of the Als5p^V326N^ protein should be resistant to the inhibitors. In fact these results were observed in cellular assays of binding to bead-bound ligand (Figure 3), and in the model biofilm study (Figures 5 and 6), the V326N mutant phenotype was mimicked by low concentrations of amyloid-perturbing dyes Congo red and thioflavin T. In keeping with this interpretation, the residual activity of Als5p^V326N^ was not inhibited by these dye concentrations, a result consistent with the remaining activity being amyloid independent.

Thioflavin T at sub-inhibitory concentrations is standardly used to monitor formation of amyloids *in vitro* [46], [47], and thioflavin T was highly useful to monitor the cell surface amyloid levels in intact cells (Figures 3, 4, and 7). Unlike wild type cells, Als5p^V326N^ cells showed minimal thioflavin T fluorescence, consistent with the idea that the mutation compromised amyloid formation. For cells expressing wild type Als5p, the fluorescence increased on aggregation, implying that cell-cell contact resulted in increased amyloid formation at the cell surface, both in the *S. cerevisiae* display model and in *C. albicans* itself, results consistent with an increase in cell surface birefringence [8], [11]. We could also use the technique to monitor peptide-induced modulation of cell surface amyloid levels (Figures 3, 4, and 7).

The finding that peptides modulated both aggregation of cells and cell surface amyloid levels in parallel was also consistent with amyloid dependence of aggregation. Amyloids are highly sequence specific, and form stacked β-sheets of identical sequences in many molecules of the same protein [22], [23]. Therefore, amyloid formation is highly sensitive to addition of amyloid-forming or amyloid-interfering peptides with sequences identical to or slightly changed from the amyloid-forming region of the protein [48], [49]. An amyloid-forming peptide rescued the *S. cerevisiae* cells expressing Als5p^V326N^, as well as increasing surface amyloid levels in cells expressing Als5p^WT^ and in *C. albicans* (Figure 4). We propose that the wild type sequence peptide reinforces amyloids with homologous sequence, and is able to form a “seed” that forces amyloid–like interactions in Als5p^V326N^. The converse experiment was also informative: excess V326N peptide inhibited amyloid formation and aggregation in cells expressing wild type adhesins. These effects were sequence-specific, since sequence-scrambled peptides of the same composition had no effects on aggregation or biofilm formation (Figure S3). Therefore, the peptide studies confirmed a specific role for amyloid formation in cellular aggregation.

That the V326N peptide inhibited aggregation and nanodomain formation in *C. albicans* also implies that Als1p or Als5p is the major adhesin being assayed. Als1p, Als3p, and Als5p have identical amyloid sequences, and Als1p is the major adhesin expressed on *C. albicans* in the yeast form [6], [50]. Therefore, the peptides would likely affect aggregation caused by Als1p as well as that caused by Als5p or Als3p.

Our results are consistent with a role for amyloid formation in causing robust aggregation of cells expressing amyloid-forming adhesins. Recent AFM studies show that the amyloid-forming sequence in Als5p mediates force-induced clustering of the adhesins on the cell surface [43]. Such clustering increases the strength of adherence because it increases the chance that ligands remain bound for long times: random dissociation of a ligand is followed by immediate rebinding to a nearby adhesin. The clustering lowers the macroscopic dissociation constant K_D_ [51]. This effect is known from immunology as antibody avidity, the increase in binding constant of intact antibody with multiple binding sites relative to the dissociation constant of a single monomeric FAb'. The clustering is absent in cells expressing Als5p^V326N^, a result consistent with amyloid formation being important for adhesin clustering (Figure 7). Like increased aggregation and amyloid formation, clustering is independent of cellular metabolism and protein synthesis [8], [39], [43], [52]. The Als adhesins cluster even though they are anchored to the cell wall polysaccharide [14], [53]. This clustering is facilitated by the length of the extended molecules, the longest of which can extend to almost 500 nm, giving a radius of gyration of almost 1 µm across the cell surface (Figure 7; Figure S5; [43]).

Thus, we find that amyloid-dependent clustering of Als5p to form adhesion nanodomains would account for increased avidity and robust aggregation (Figure 7Q). Amyloid formation at the cell surface is accompanied by conformational shifts in pre-existing cell surface proteins, and is co-temporal with development of robust aggregation. Three different modulators of amyloid forming ability prevented formation of thioflavin fluorescent amyloid nanodomains and abrogated strong aggregation: namely mutation, chemical perturbants, and an amyloid-disrupting peptide. Furthermore, adhesion was enhanced after exogenous application of an amyloid-forming Als5p homologous sequence peptide. The enhancement was accompanied by increased amyloid fluorescence, and peptides with scrambled sequences had no effect. Thus all of our results are consistent with amyloid formation itself being an essential part of cellular aggregation. We cannot think of other mechanisms consistent with the data.

Formation of amyloid-dependent adhesion nanodomains has broad implications as a mechanism for yeast cell-cell adhesion in general, as well as in mats and biofilms [15], [42]. Amyloid sequences are present in most yeast adhesins [11]. Furthermore, for *C. albicans* Als proteins, and *S. cerevisiae* Flo1p and Muc1p/Flo11p, adhesins, activity is inhibited by amyloid-perturbing dyes. These results, together with our discovery of the effects of specific sequence peptides promise new approaches to understanding and manipulation of cell-cell interactions.

## Materials and Methods

### Strains and media

*C. albicans* strain Day 286 was a gift from J. Rauceo (John Jay College, CUNY) and was grown in YPED with 80 mg/L uridine at 30°C. *S. cerevisiae* strain W303-1B (Rodney Rothstein, Columbia U.) was grown in CSM with galactose at 24°C. Cells harboring empty vector (pJL1-EV), or expressing Als5p^WT^ or Als5p^V326N^ were grown in CSM with galactose lacking Trp.

### Generation of V5-tagged Als5p^WT^ and Als5p^V326N^

V5-tagged Als5p^WT^ was generated by restriction digestion of pGK114 with *BamHI* and *XhoI* which released the Als5p sequence from the vector backbone [39]. This backbone was then ligated to: an oligonucleotide, which contains sequences for the invertase secretion signal, V5 epitope tag, flanked by a 5′*-Bam*HI and 3′-*Not*I restriction sites [54]. The coding region of Als5p between a 5′ *Not*I site and a 3′*Xho*I site was generated by PCR and was ligated to the modified vector to make pJL1. The resulting construct was verified by sequencing (GeneWiz, South Plainfield, NJ).

Als5p^V326N^ was generated by digestion of pGK114 with *Sph*I and *Ale*I to generate a 363bp fragment at nucleotide position 1242 to 1605, containing the target sequence to be mutated. This fragment was subcloned into pGEM-T vector and mutagenized using Quickchange (Agilent Technologies, Santa Clara, CA) with mutagenic primer 5′-GAA TAG TGA TGC CGG ATC TAA CGG TAT TAA CAT TGT TGC TAC AAC TAG AAC AGT TAC AGA CAG-3′. The correct mutation was verified by sequencing. The mutated fragment was released from the vector with the same enzymes used in its generation, and placed into the corresponding position of pJL1. The resulting product, pJL1^V326N^ was verified by sequencing to determine the presence of the full-length Als5p^V326N^.

pJL-EV was produced by restriction digestion of pJL1 with *Bam*HI and *Xho*I and ligating in the multiple cloning site from p414 (ATCC, Manassas, VA). The pJL plasmids were transformed into *S. cerevisiae* strain W303-1B.

### Cell-bead assay

Cell aggregation was observed using a previously described method with minor modifications [8]. Briefly, M-280 tosyl-activated magnetic Dynabeads (Invitrogen, Carlsbad, CA) were coated overnight at 37°C with 1 mg/ml heat-denatured BSA according to the manufacturer's protocol. Cells were washed and resuspended in 10 mM Tris-HCl, 1 mM EDTA buffer, pH 7.0 (TE). Beads, 1×10^6^, were added to 1×10^8^ cells in 13×100 mm glass test tubes and the suspension was shaken at 200 rpm at 24°C 30-45 minutes. Adherent cells were separated and washed over a magnet, resuspended in TE buffer and observed by microscopy. Thioflavin T fluorescence was excited at 425 nm and monitored at 510 nm.

### Polystyrene cell adhesion assay

Adhesion to polystyrene was assayed using a protocol previously described [42] with the following modifications. Cells were resuspended in TE alone or with dyes at 1×10^7^ cells/ml. Cell suspension, 100 µl/well, was added to a polystyrene non-tissue culture plate with 96 wells. Each sample was done in triplicate. The cells adhered to the surface for 1.5 hours at room temperature. The polystyrene surface was washed with TE, sterile liquid media was added and the adherent cells were incubated overnight at 30°C. The next day the non-adherent cells were washed away with TE. Adherent cells were stained with crystal violet and observed by microscopy and imaged. Adhesion was quantified by solubilization of the crystal violet in 10% SDS for 1.5 hours and measuring the absorbance at 570 nm.

### Atomic force microscopy

AFM measurements were performed at room temperature (20°C) in buffered solutions (sodium acetate; pH 4.75), using a Nanoscope IV Multimode AFM (Veeco Metrology Group, Santa Barbara, CA) and oxide sharpened microfabricated Si_3_N_4_ cantilevers (Microlevers, Veeco Metrology Group). Cells were immobilized by mechanical trapping into porous polycarbonate membranes (Millipore), with a pore size similar to the cell size. After filtering a concentrated cell suspension, the filter was gently rinsed with buffer, carefully cut (1 cm ×1 cm), attached to a steel sample puck (Veeco Metrology Group) and the mounted sample was transferred into the AFM liquid cell while avoiding dewetting. The spring constants of the cantilevers were measured using the thermal noise method (Picoforce, Veeco Metrology Group), yielding values ranging from 0.008 to 0.021 N/m. All force measurements were recorded with a loading rate of 10,000 pN/s. AFM tips were functionalized with anti-V5 antibodies or Als5p^1-431^ protein as described earlier [43], [44].

## Supporting Information

Figure S1 **ELISA assays of binding of Als5p^V326N^ substitution proteins.** The upper graph shows binding of increasing concentrations of proteins to different concentrations of fibronectin. The lower graph denotes binding of the proteins to polystyrene. The constructs shown are Ig-T-TR, wildtype (•) and V326N (▾), and Ig-T wildtype (○) and V326N (Δ). The protein concentration is 2.3±0.7μM for the fibronectin binding. The assays were carried out as previously described [11].
(TIF)

Figure S2 **Far UV Circular Dichroism spectra of Als5p ^1-664^ protein.** Wildtype protein is represented by the solid line and the V326N substation by the dashed line at 20°C.
(TIF)

Figure S3 **Effects of scrambled V326N (VITGVTNIRTSVA) and wild type peptide (VITGNTNIRTSVA) on cellular aggregation.** *S. cerevisiae* expressing no Als5p (EV), Als5p^V326N^, Als5p^WT^, or *C. albicans* were aggregated in the absence and presence of 2µg/ml scrambled wild type (S-wild type) or 200 µg/ml scrambled peptide (S-V326N). The diameter of the beads is 2.8 µm, and all images are at the same magnification.
(TIF)

Figure S4 **Amyloid binding dye Congo red reduces binding and aggregation on polystyrene biofilms.** *C. albicans* or *S. cerevisiae* expressing Als5p^WT^, Als5p^V326N^, or no Als5p (EV) adhered to a polystyrene surface in the absence and presence of 100 nM or 300 µM Congo red for 1.5 h. Adherent cells were grown overnight, stained with 1% crystal violet and imaged.
(TIF)

Figure S5 **Detection and unfolding of single Als proteins in** ***C. albicans.*** (A) Principle of the single-molecule detection experiment. *C. albicans* cells are probed, in buffer, using AFM tips derivatized with Als5p^1-431^. (B) Force extension curves obtained by stretching ALS proteins showed periodic features reflecting the sequential unfolding of the TR domains. (C) Plot of the rupture distances as a function of the number of unfolded TR.
(TIF)
