# Supplementary figures and images for: A Role for Amyloid in Cell Aggregation and Biofilm Formation
Source: PLoS One. 2011 Mar 8;6(3):e17632. doi: 10.1371/journal.pone.0017632 (PMC3050909; doi:10.1371/journal.pone.0017632)

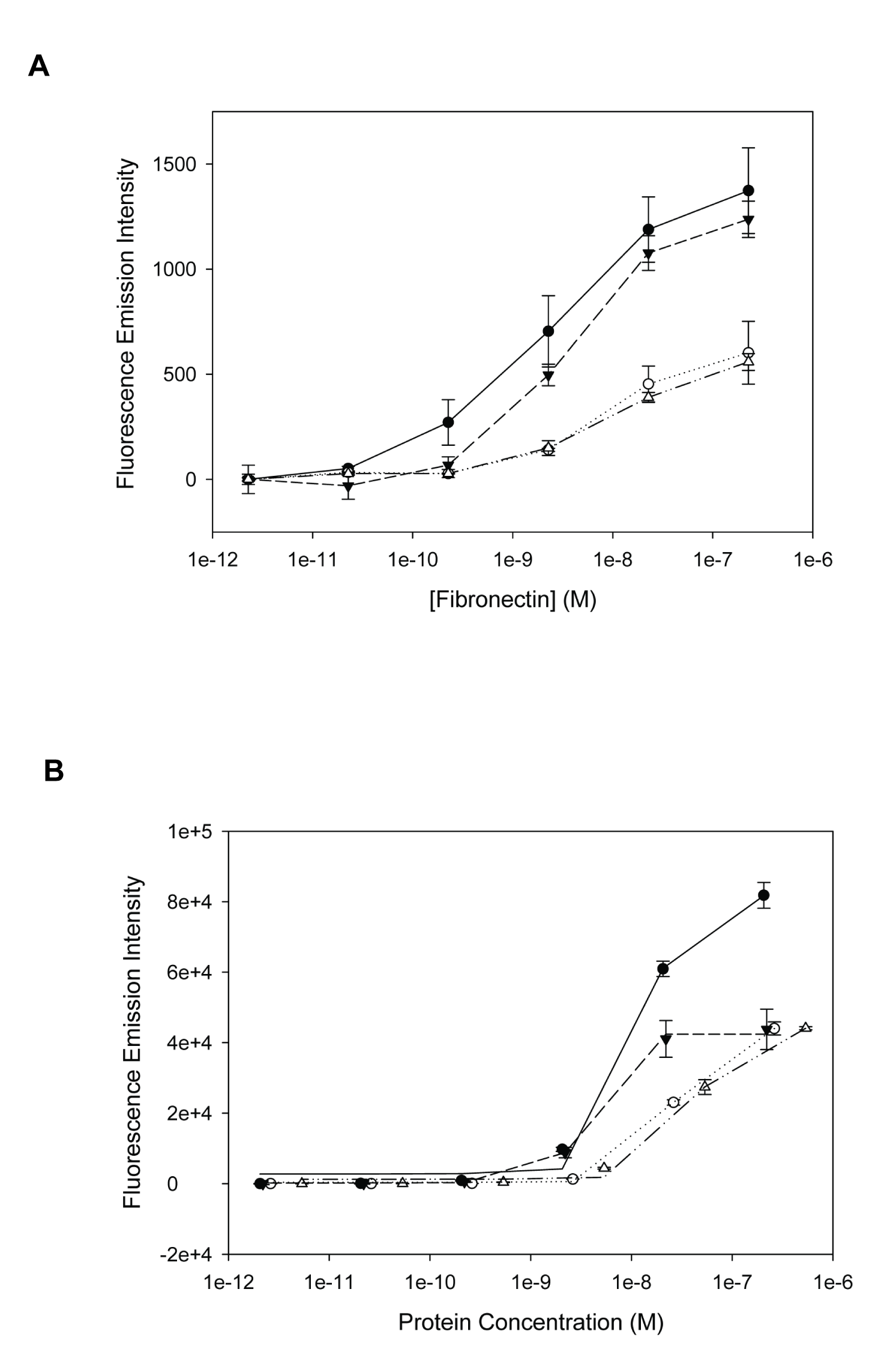

Supplement: Figure S1 — ELISA assays of binding of Als5pV326N substitution proteins. The upper graph shows binding of increasing concentrations of proteins to different concentrations of fibronectin. The lower graph denotes binding of the proteins to polystyrene. The constructs shown are Ig-T-TR, wildtype (•) and V326N (▾), and Ig-T wildtype (○) and V326N (Δ). The protein concentration is 2.3±0.7μM for the fibronectin binding. The assays were carried out as previously described [11]. (TIF) [file pone.0017632.s001.tif]

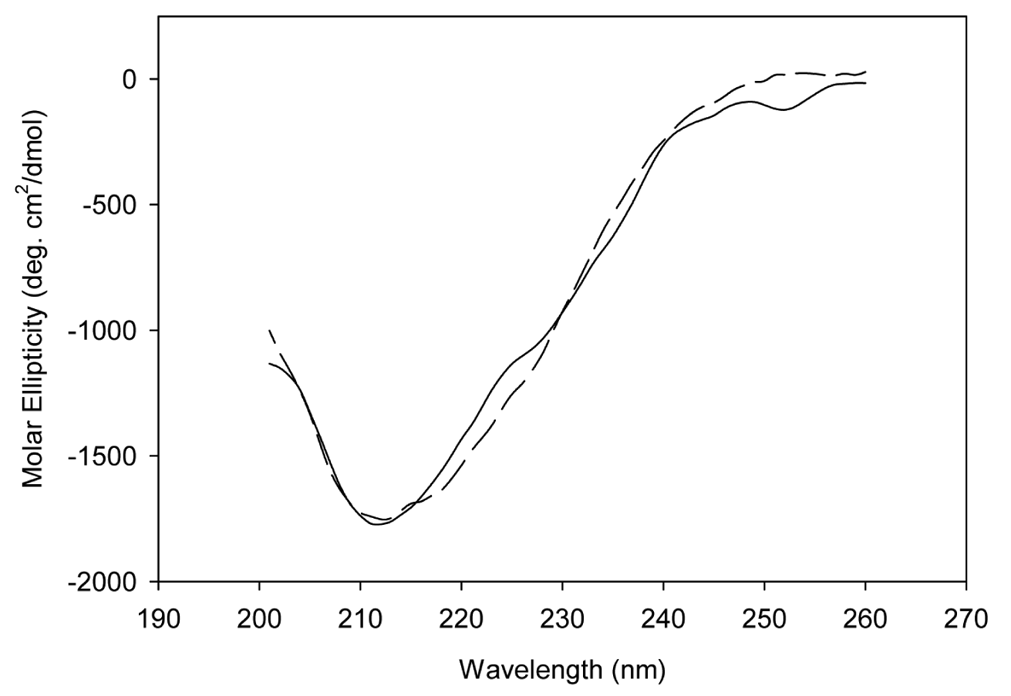

Supplement: Figure S2 — Far UV Circular Dichroism spectra of Als5p 1-664 protein. Wildtype protein is represented by the solid line and the V326N substation by the dashed line at 20°C. (TIF) [file pone.0017632.s002.tif]

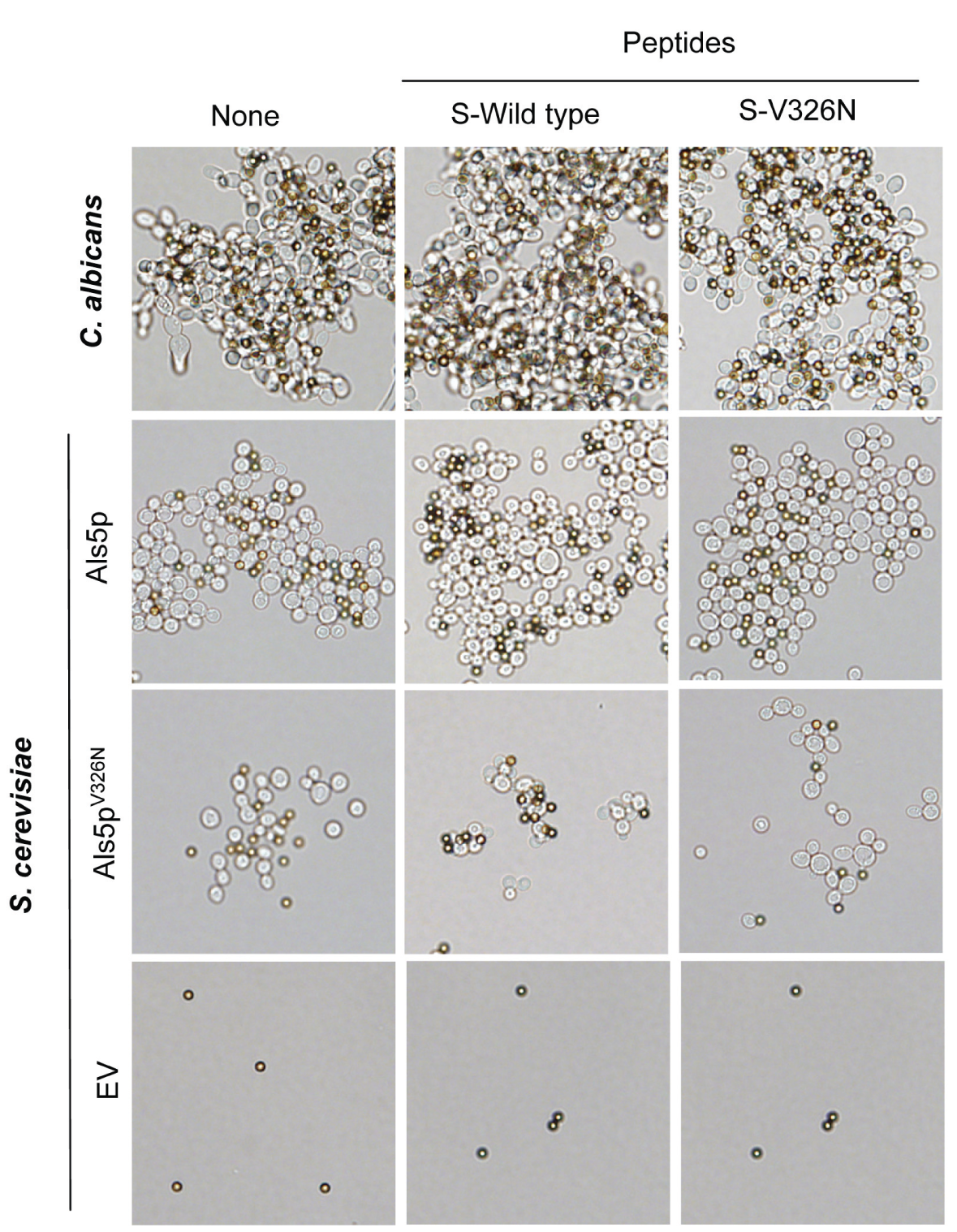

Supplement: Figure S3 — Effects of scrambled V326N (VITGVTNIRTSVA) and wild type peptide (VITGNTNIRTSVA) on cellular aggregation. S. cerevisiae expressing no Als5p (EV), Als5pV326N, Als5pWT, or C. albicans were aggregated in the absence and presence of 2µg/ml scrambled wild type (S-wild type) or 200 µg/ml scrambled peptide (S-V326N). The diameter of the beads is 2.8 µm, and all images are at the same magnification. (TIF) [file pone.0017632.s003.tif]

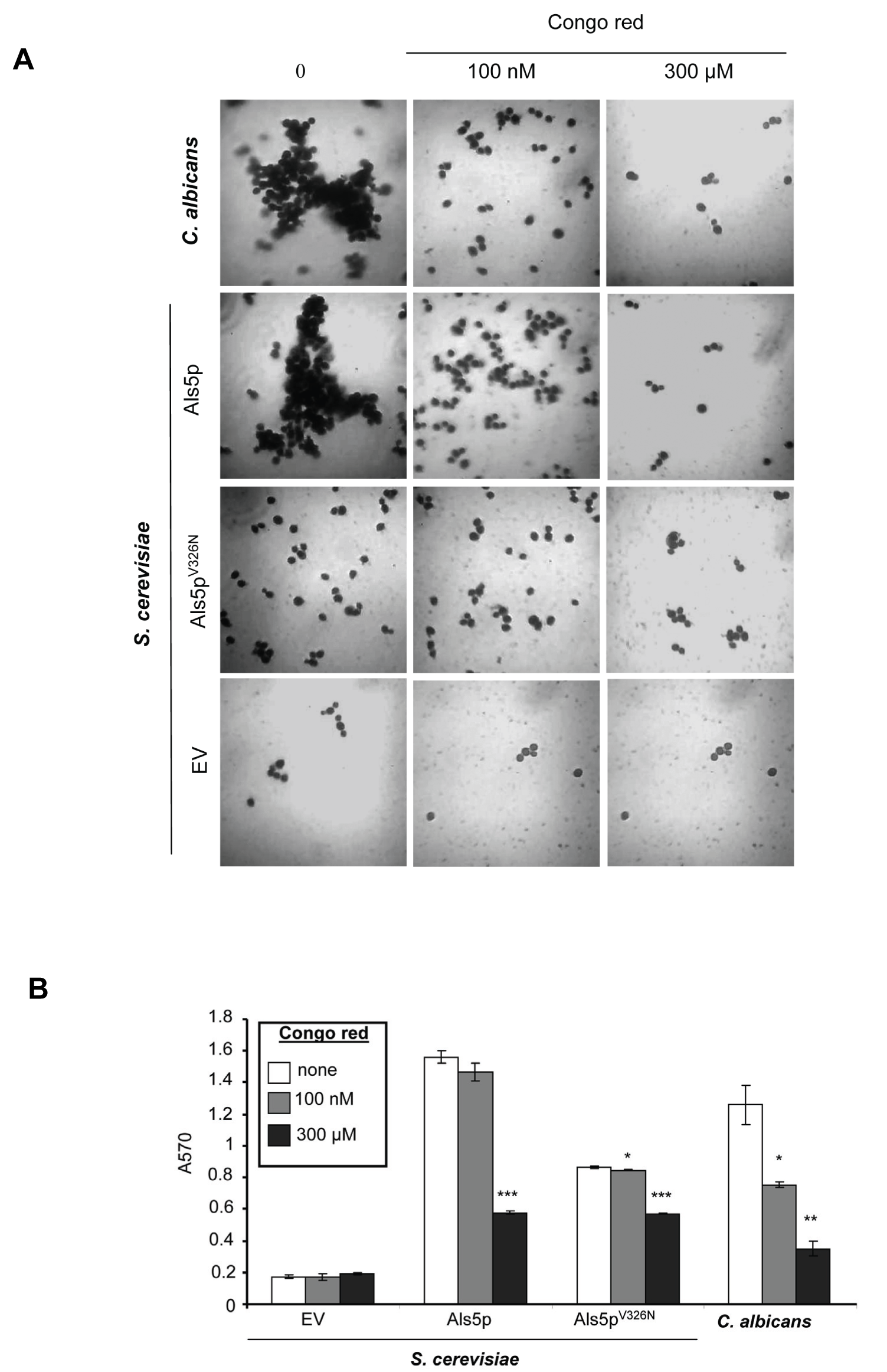

Supplement: Figure S4 — Amyloid binding dye Congo red reduces binding and aggregation on polystyrene biofilms. C. albicans or S. cerevisiae expressing Als5pWT, Als5pV326N, or no Als5p (EV) adhered to a polystyrene surface in the absence and presence of 100 nM or 300 µM Congo red for 1.5 h. Adherent cells were grown overnight, stained with 1% crystal violet and imaged. (TIF) [file pone.0017632.s004.tif]

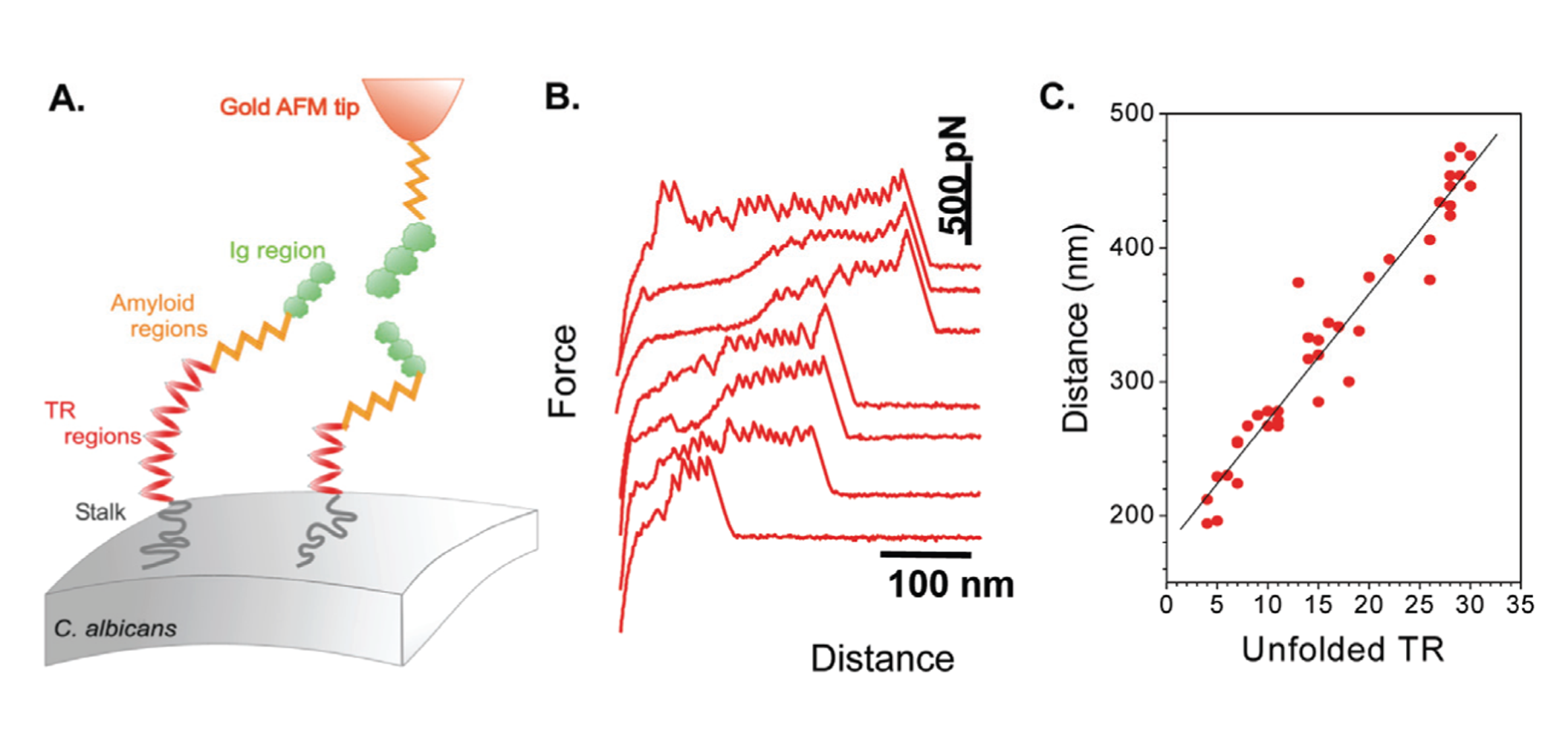

Supplement: Figure S5 — Detection and unfolding of single Als proteins in C. albicans. (A) Principle of the single-molecule detection experiment. C. albicans cells are probed, in buffer, using AFM tips derivatized with Als5p1-431. (B) Force extension curves obtained by stretching ALS proteins showed periodic features reflecting the sequential unfolding of the TR domains. (C) Plot of the rupture distances as a function of the number of unfolded TR. (TIF) [file pone.0017632.s005.tif]
